# Rev1 *wbdR* tagged vaccines against *Brucella ovis*

**DOI:** 10.1186/s13567-019-0714-3

**Published:** 2019-11-15

**Authors:** Beatriz Aragón-Aranda, María Jesús de Miguel, Estrella Martínez-Gómez, Amaia Zúñiga-Ripa, Miriam Salvador-Bescós, Ignacio Moriyón, Maite Iriarte, Pilar M. Muñoz, Raquel Conde-Álvarez

**Affiliations:** 1 Instituto de Salud Tropical (ISTUN), Instituto de Investigación Sanitaria de Navarra (IdiSNA) and Dpto. de Microbiología y Parasitología, Universidad de Navarra, c/Irunlarrea 1, 31008 Pamplona, Spain; 2 Unidad de Producción y Sanidad Animal, Instituto Agroalimentario de Aragón-IA2 (CITA-Universidad de Zaragoza), Av. Montañana 930, 50059 Saragossa, Spain

## Abstract

Sheep brucellosis is a worldwide extended disease caused by *B. melitensis* and *B. ovis*, two species respectively carrying smooth or rough lipopolysaccharide. Vaccine *B. melitensis* Rev1 is used against *B. melitensis* and *B. ovis* but induces an anti-smooth-lipopolysaccharide response interfering with *B. melitensis* serodiagnosis, which precludes its use against *B. ovis* where *B. melitensis* is absent. In mice, Rev1 deleted in *wbkC* (*Brucella* lipopolysaccharide formyl-transferase) and carrying *wbdR* (*E. coli* acetyl-transferase) triggered antibodies that could be differentiated from those evoked by wild-type strains, was comparatively attenuated and protected against *B. ovis*, suggesting its potential as a *B. ovis* vaccine.

## Introduction

Brucellosis is one of the most common zoonoses worldwide, causing significant loss to animal production and severely affecting human health [1]. The etiological agents of brucellosis are Gram-negative bacteria of the genus *Brucella.* This genus includes several zoonotic species among which *B. abortus* preferentially infects cattle, *B.* *suis* swine and wild-life and *B. melitensis* goats and sheep [2]. Sheep can also be infected by *B. ovis*, a non-zoonotic species [2]. These species are smooth (S) or rough (R), depending on the presence or absence respectively of O-polysaccharide (O-PS) chains in the outer membrane lipopolysaccharide (LPS). Whereas *B. abortus*, *B. melitensis* and *B. suis* carry a S-LPS, *B. ovis* is a naturally a R species [2].

*B. ovis* infection causes genital lesions and reduced fertility constituting one of the most important causes of reproductive failure in sheep [3]. Animal vaccination is the most suitable method for controlling brucellosis in areas with moderate to high prevalence of the disease. Currently, no specific vaccines against *B. ovis* infection are available, but the S live attenuated *B. melitensis* Rev1 vaccine, widely used for vaccination against ovine and caprine brucellosis caused by *B. melitensis*, is also effective against *B. ovis* [4]. However, Rev1 is virulent in humans, induces abortions when used in pregnant animals [4] and is resistant to streptomycin, an antibiotic of choice for brucellosis treatment [5]. While these problems can be solved by using appropriate vaccination strategies and biosafety precautions [4, 6], Rev1 also induces a strong antibody response to the O-PS section of S-LPS [7] thus hampering differentiation between true infected and vaccinated animals (DIVA problem) in the routine diagnosis of *B. melitensis*. Because of this, Rev1 is banned in the countries where *B. melitensis* has been eradicated, resulting in a subsequent increase in *B. ovis* infections in sheep. Since *B. ovis* is naturally R, some attempts to circumvent the problems associated with Rev1 vaccination in *B. melitensis*-free areas have been based on the use of *B. ovis*. Accordingly, some investigations have examined subcellular vaccines carrying *B. ovis* fractions rich in envelope components in lipid-muramyl dipeptide or nanoparticle adjuvants [8, 9]. However, these formulations either provide less protection than Rev1 or become too costly. Also, *B. ovis* mutants in LPS core genes [10] have been explored with promising results. Similarly, a mutant in putative ABC transporter encapsulated in alginate has been proposed as *B. ovis* vaccine [11, 12]. Yet, industrial production of these vaccines would require solving the problem posed by *B. ovis* CO_2_-dependence [13] with the subsequent reassessment of their properties. Moreover the ABC transporter mutant requires encapsulation [12]. R mutants of S *Brucella* species (i.e., the so-called R vaccines) are more easily produced and, as they lack the O-PS, are often assumed to solve the Rev1 DIVA problem. However, R vaccines still interfere in S-LPS ELISA [14–16] because of the cross-reactivity with the core epitopes shared by the S and R-LPS or, in the *wzm*/*wzt* and related *B. melitensis* 115 spontaneous R mutants, presence of a cytoplasm O-PS precursor [17–19]. Another approach was to delete protein BP26. However, whereas the BP26-deleted Rev1 provides protection against *B. ovis*, the ancillary BP26 ELISA lacks adequate diagnostic sensitivity [20, 21]. More recently, a Rev1 construct expressing the green fluorescent protein as a tagging antigen has been proposed. However, this method requires the simultaneous injection of this protein and a booster injection to trigger antibody persisting in the ancillary ELISA‐green fluorescent protein DIVA test [22].

The aim of this research is to investigate an alternative to circumvent the serological diagnosis problems caused by the Rev1 vaccine while keeping its good attenuation and protection characteristics. Our approach differs from those summarized above in that, instead of deleting S *Brucella* antigens or epitopes or introducing a foreign antigen, we have modified a *Brucella* immunodominant antigen. For this purpose, we applied to Rev1 the strategy proposed by Martínez-Gómez et al. [23] to modify the epitopic structure of *Brucella* S-LPS by substituting the N-formyl-perosamine of the O-PS by N-acetyl-perosamine. We present here the experiments carried out in the mouse model as a first step to investigate the validity of this approach.

## Materials and methods

### Bacterial strains and growth conditions

The bacterial strains and plasmids used are listed in Additional file 1. For construction of mutants, *B. melitensis* 16 M and Rev1 strains were grown at 37 °C in tryptic soy broth (TSB, Biomérieux, Marcy l’Etoile, France) or in this medium supplemented with agar (TSA, Pronadisa, Conda, Spain). *B. ovis* strains were grown at 37 °C in TSB supplemented with 0.5% yeast extract (Pronadisa, Conda, Spain) and 5% fetal bovine serum (TYSB-S) or this medium supplemented with agar (TYSA-S). For the studies in mice, vaccines and challenge strain were grown in Blood Agar Base (BAB, Oxoid) or BAB-S (supplemented with 5% fetal bovine serum). Where needed, media were supplemented with 5% sucrose (Sigma), diaminopimelic acid (DAP; 1 mM), 0.2% activated charcoal (Sigma), kanamycin (Km) at 50 µg/mL, chloramphenicol (Cm) at 20 µg/mL, ampicillin (Amp) at 100 µg/mL, polymyxin (Pmx) at 1.5 µg/mL or streptomycin (Strp) at 2.5 µg/mL. All strains were stored at −80 °C in skimmed milk (Scharlau, Barcelona, Spain) or TYSB-7% dimethylsulfoxide (DMSO).

### DNA manipulations

Plasmid and chromosomal DNA were extracted with Q1Aprep^®^ spin Miniprep Kit (Qiagen GmbH, Hilden, Germany) and Ultraclean Microbial DNA Isolation Kit (Mo Bio Laboratories), respectively. When needed, DNA was purified from agarose gels using a QIAquick Gel extraction kit (Qiagen). DNA sequencing was performed by “Servicio de Secuenciación del Centro de Investigación Médica Aplicada” (Pamplona, Spain). Primers were synthesized by Sigma-Genosys Ltd. (Haverhill, United Kingdom).

### Construction of mutants

For the construction of Rev1::Tn7*wbdR*Km^R^ mutants, we used the plasmid pYRI-27 (pUC18R6KT-miniTn7T-Km-P*wbdR*) described in Martínez-Gómez et al. [23]. The acquisition of this vector by *Brucella* after tetra-parental mating with conjugative *E. coli* S17.1 λpir and *E. coli* HB101 (pRK2013) and *E. coli* SM10 λpir (pTNS2) was selected by Km and Pmx resistance. The correct insertion and orientation of the miniTn7 carrying *wbdR* was examined by PCR as previously described [23].

To obtain a *wbdR* construct with no Km resistance (Rev1::Tn7*wbdR*), we used the plasmid pRCI-65 (pNPTS138Cm^R^Δ*Km*) described in Martínez-Gómez et al. [23]. This suicide plasmid containing the *Km*R deletion allele was transformed into *E. coli* β2150, a diaminopimelic acid (DAP) auxotrophic donor strain, to avoid the use of antibiotic during the conjugation process [24] and transferred into Rev1::Tn7*wbdR*Km^R^ by conjugation. The integration of the suicide vector and disruption of the target gene were selected by Km sensitivity and confirmed by PCR using oligonucleotides *KmR*-F1 and *KmR*-R4 (Additional file 2).

Rev1::Tn7*wbdR*Δ*wbkC* was constructed in a similar way. The suicide plasmid pYRI-31 (pJQKmΔ*wbkC*) [23] was used to delete the *wbkC* gene of Rev1::Tn7*wbdR* by allelic change. The resulting mutator plasmid was introduced in Rev1::Tn7*wbdR* by conjugation using the *E. coli* β2150. The loss of the plasmid concomitant with the deletion of *wbkC* gene was checked by PCR using oligonucleotides *wbkC*-F1 and *wbkC*-R4 (Additional file 2).

The strain *B. ovis* PA-KmR, used as challenge in mouse experiments (see below), was obtained using the modified miniTn7 site-specific integration vector technology [25, 26].

### Characterization of the mutants

Mutants were characterized by the standard *Brucella* typing procedures described in Alton et al. [27]: colonial morphology, urease, susceptibility to thionine blue, fuchsine and safranine, acriflavine agglutination, crystal violet dye exclusion test (S/R colony morphology), agglutination with anti-A and anti-M monospecific sera, and sensitivity to phages. For co-agglutination, bacteria resuspended in 25 µL of saline on a glass slide were mixed with an equal amount staphylococci sensitized with anti-Formyl-Acetyl, anti-Acetyl and anti-Formyl sera, as previously described [23].

### LPS extraction

LPS from Rev1 and Rev1 *wbdR* tagged was obtained by the proteinase-K sodium dodecyl sulfate (SDS) protocol [28, 29] with some modifications. Briefly, cells inactivated with phenol were suspended in 2% SDS-60 mM Tris–HCl buffer (pH 6.8), heated at 100 °C for 10 min and treated with proteinase K (60 µL of a 2.5 mg/mL stock per each mL of suspension, 3 h at 55 °C). This was followed by two consecutive precipitations with 3 volumes of methanol with 1% sodium acetate-saturated methanol at −20 °C. After centrifugation, pellets were resuspended by sonication in 3 mL of 60 mM HCl-Tris (pH 6.8), digested with nucleases and treated again with proteinase K (3 h at 55 °C). After a third precipitation, the pellet containing LPS was recovered in 1 mL of distilled water and frozen.

### SDS-PAGE and Western blots

Samples were mixed 1:1 with Sample buffer 2x (Bio-Rad), heated at 100 °C for 10 min, and analyzed in 15% polyacrylamide gels in Tris–HCl-glycine and stained by the periodate-alkaline silver method [30]. For Western blots, LPS were analyzed in SDS-PAGE gel (12% polyacrylamide) (see above) and electrotransferred onto nitrocellulose blotting sheets (Amersham- GE Healthcare Life Scientific, Germany; 0.45 µm pore size). The polyclonal sera used were the following: Anti-S *Brucella* serum (polyclonal serum from a rabbit infected with *B. melitensis* 16 M and bled at day 45), Formyl-Acetyl serum and Acetyl serum. These last two sera were obtained by the method described in Martínez-Gómez et al. [23].

### Multiplex PCR

To discriminate between Rev1 *wbdR* tagged strains and all known *Brucella* species and vaccine strains, we designed a pair of primers based on the *wbdR* gene and added them to the 8 pairs of primers used in the Bruce-ladder assay [31]. These new primers consisted of 5´-TGATGTTTTGGCAGGAAAGA-3′ (*wbdR* forward) and 5′-TAGCCCCAGGAGCAAATGTA-3′ (*wbdR* reverse). We designed them with a Tm similar to that of the other primers used in the multiplex PCR and amplifying a band of 347 bp that did not prevent the visualization of the other specific bands of species or vaccine strains.

### Studies in mice: virulence and protection

Seven-week-old female BALB/C mice (ENVIGO, Harlan) were kept in cages in BSL-3 facilities (ES/31-2010-000132) with water and food *al libitum*. Procedures were in accordance with the current European (directive 86/609/EEC) and Spanish (RD 53/2013) legislations, supervised by the corresponding Ethical Committee for Animal Experimentation and authorised by Aragón (reports No. 2014-20 and 2014-21) and Navarra (CEEA 045/12) Governments. For virulence assessment, 10-mice groups were inoculated intraperitoneally (IP) with 1 × 10^5^ or 1 × 10^8^ colony forming units (CFU) of the corresponding strain and mean CFU values per spleen were determined at 1 (*n* = 5) and 5 (*n* = 5) weeks after inoculation as described elsewhere [32]. To evaluate the protective efficacy of Rev1::Tn7*wbdR*Δ*wbkC* vaccine, 5-mice groups were vaccinated subcutaneously (SC) with two different doses (1 × 10^5^ and 1 × 10^8^ CFU/mouse). Mice (*n* = 5) inoculated SC with the Rev1 reference vaccine (1 × 10^*5*^ CFU/mouse) or sterile Buffered Saline (BSS; 0.85% NaCl, 0.1% KH_2_PO_4_, 0.2% K_2_HPO_4_; pH 6.85) were used as effective-vaccine and unvaccinated controls, respectively. Four weeks after vaccination, mice were challenged IP with 5 × 10^6^ CFU/mouse of *B. ovis* PA-KmR and the mean CFU/spleen values of this strain were determined 2 weeks after. Inocula were prepared by harvesting the BAB or BAB-S grown bacteria in sterile BSS, adjusting spectrophotometrically (600 nm) the bacterial suspension and making proper serial tenfold dilutions. Mice were inoculated with 0.1 mL and doses were retrospectively assessed by plating inocula countable dilutions. Differentiation between challenge and vaccine strains was achieved by plating on BAB supplemented with streptomycin and BAB-S supplemented with Km (incubated in CO_2_ atmosphere). Results were expressed as the mean log_10_ CFU/spleen ± SD (*n* = 5) of the corresponding mutant or challenge strain and units of protection were calculated by subtracting the mean log_10_ CFU values of the vaccine group from those of unvaccinated controls. Statistical comparisons were made by one-way ANOVA and Fisher’s Protected Least Significant Differences (PLSD) post hoc tests. The adequate virulence of *B. ovis* PA-KmR challenge strain was proved in a previous experiment in BALB/c mice by IP infection (5 × 10^5^ CFU/mouse) and bacterial spleen counting (in BAB-S and BAB-S supplemented with Km) 3 and 8 weeks later. This strain showed identical multiplication to that of *B. ovis* PA (not shown).

### Enzyme-linked immunosorbent assay (iELISA)

Serum antibodies of mice inoculated with Rev1 (parental strain), Rev1::Tn7*wbdR*, Rev1::Tn7*wbdR*Δ*wbkC* and *B. melitensis* 16 M (as control) were analyzed using an iELISA with wild-type (N-formyl-perosamine) or modified (N-acetyl-perosamine) S-LPS as antigens. For this, 96-well plates (Thermo Scientific, Waltham, MA, USA) were coated by overnight incubation at 4 °C with *B. melitensis* 16 M or Ba::Tn7*wbdR*Δ*wbkC* S-LPS at 2.5 µg/mL or 5 µg/mL, respectively, in PBS. Plates were washed with PBS-0.05% Tween 20 (and incubated with serial dilutions of sera at 37 °C for 30 min. After washing, antibodies were detected with peroxidase-labeled protein G [19] and 2,2′-azino-bis (3-ethylbenzthiazoline-6-sulphonic acid) (ABTS)-H_2_O_2_. After 15 min, colorimetric reactions were read at 405 nm (Multiescanex, Thermo Scientific, Waltman, MA, USA). Results are expressed as optical density (O.D.) values of tested sera after subtracting the O.D. value of the negative control (blank well) in the same plate.

## Results

### Insertion of *wbdR* into Rev1 genome modifies the epitopic structure of the vaccine

**Figure 1:**
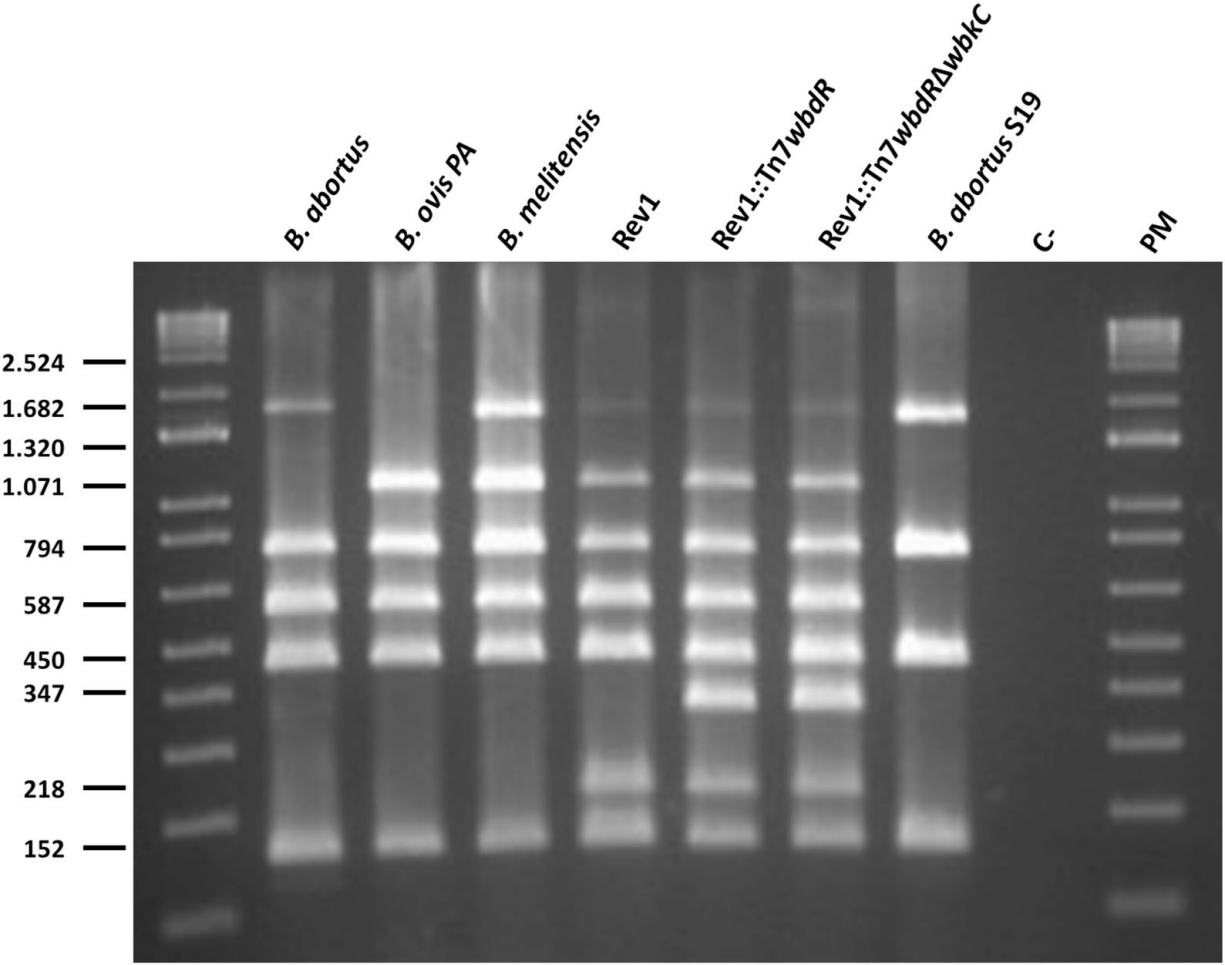
***wbdR* tagged vaccines can be identified by a modified multiplex PCR assay.** Identification and differentiation of *Brucella* species, classical vaccines and Rev1 *wbdR* tagged vaccines by a modified Bruce-Ladder [31] that includes a new pair of primers based on the *wbdR* gene that amplify a band of 347 bp specific for tagged vaccines. C–(H_2_O) and PM (1 Kb plus DNA ladder) used as molecular size marker.

To obtain a tagged Rev1 vaccine, we followed two strategies. First, we obtained Rev1::Tn7*wbdR* inserting *wbdR* in the chromosome II of Rev1 vaccine using the previously described Tn7 methodology [8]. Second, we constructed a *wbdR* tagged Rev1 lacking N-formyl-perosamine in its O-PS (Rev1::Tn7*wbdR*Δ*wbkC)* deleting *wbkC* (the formyl transferase gene) from Rev1::Tn7*wbdR.* As expected, these constructs could be differentiated from Rev1, vaccine *B. abortus* S19, or representative strains of wild-type *B. abortus*, *B. melitensis* and *B. ovis* by PCR using the Multiplex-PCR combining Bruce-ladder and *wbdR* specific primers (Figure 1).

Rev1::Tn7*wbdR* and Rev1::Tn7*wbdR*Δ*wbkC* were identical to the parental strain in colony morphology, growth, oxidase and urease tests and dye sensitivity (Additional file 3). Consistent with previous results in virulent *B. melitensis* 16 M [23], Rev1::Tn7*wbdR* and Rev1::Tn7*wbdR*Δ*wbkC* did not agglutinate with acriflavine and excluded crystal violet, properties characteristic of strains expressing S-LPS. On the other hand, both *wbdR* tagged strains were sensitive to phage R/C, a phage specific for the O-PS-lacking R brucellae [27], a phenotype not observed previously on *wbdR* constructs of virulent strains.

**Figure 2:**
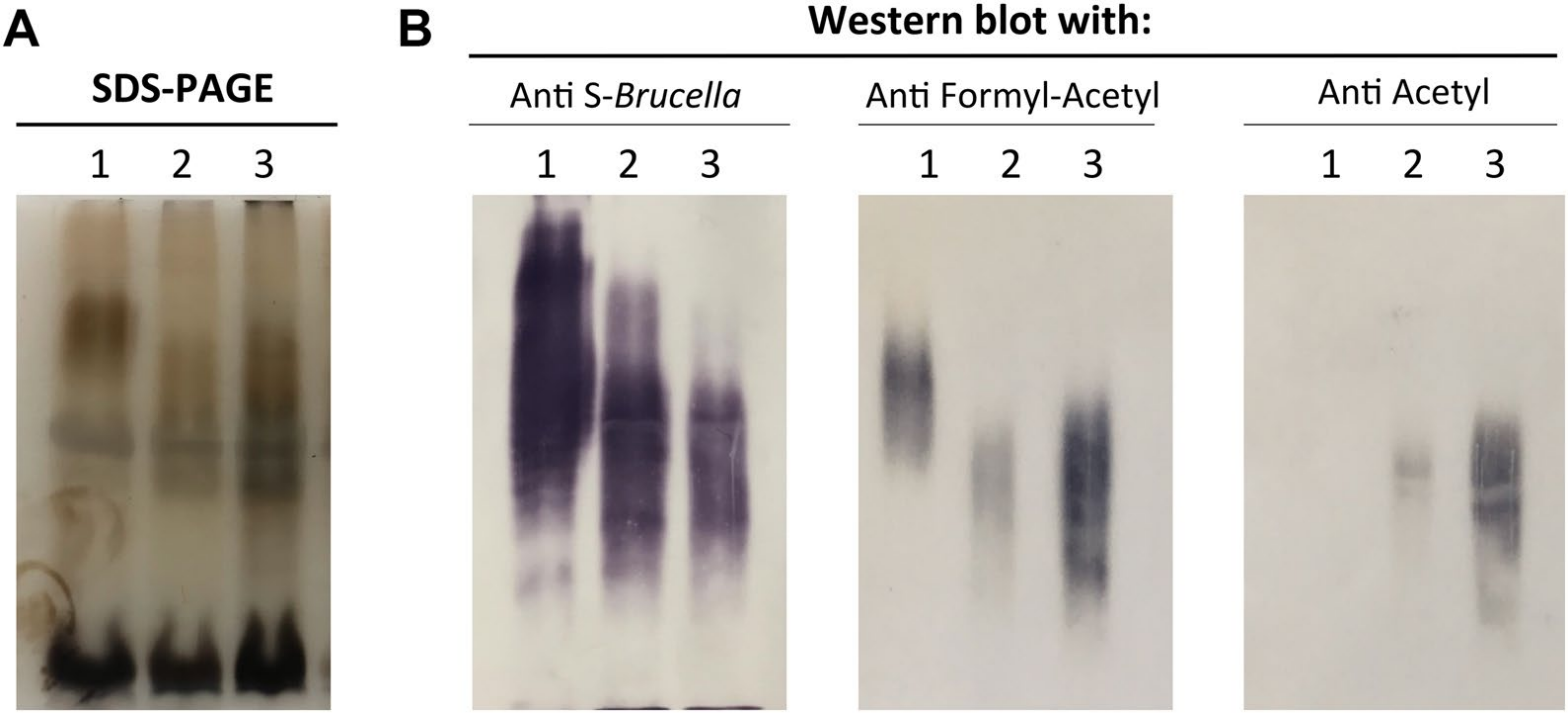
**Insertion of** ***wbdR* into Rev1 genome modifies the epitopic structure of the vaccine.** **A** SDS-PAGE electrophoresis-silver staining and **B** Western blot analyses of SDS-proteinase K extracts of (1) Rev1, (2) Rev1::Tn7*wbdR,* and (3) Rev1::Tn7*wbdR*Δ*wbkC*.

The LPS extracts of Rev1::Tn7*wbdR* and Rev1::Tn7*wbdR*Δ*wbkC* contained the typical R-LPS (i.e. low molecular weight) and S-LPS (higher molecular weight) fractions present in the parental strain (Figure 2). However, as observed before for their *wbdR* tagged *B*. *melitensis* and *B. abortus* counterparts [23], the S-LPS fraction of the *wbdR* constructs displayed an apparent average molecular weight lower than those of the parental strains (Figure 2A). To analyze possible epitopic changes associated with *wbdR* tagging, whole cells and LPS extracts were tested by co-agglutination and Western blot, respectively, using immune sera specifically recognizing O-PS carrying only N-formyl-perosamine (anti-Formyl), both N-formyl-perosamine and N-acetyl-perosamine (anti-Formyl-Acetyl) or only N-acetyl-perosamine (anti-Acetyl) [23]. Rev1::Tn7*wbdR* co-agglutinated with staphylococci sensitized with anti-Formyl-Acetyl, anti-Acetyl and anti-Formyl sera showing that its O-PS contained N-acetyl and N-formyl-perosamine. In contrast, Rev1::Tn7*wbdR*Δ*wbkC* did not co-agglutinate with anti-Formyl serum, while keeping the anti-Formyl-Acetyl and anti-Acetyl reactivity (Additional file 4). Western blots with proteinase-K extracted S-LPS (Figure 2B) confirmed the epitopic changes and demonstrated that the differences in apparent average molecular weight observed by SDS-PAGE (Figure 2A) corresponded in fact to O-PS heterogeneity.

### *wbdR* tagging decreases the residual virulence of Rev1

**Figure 3:**
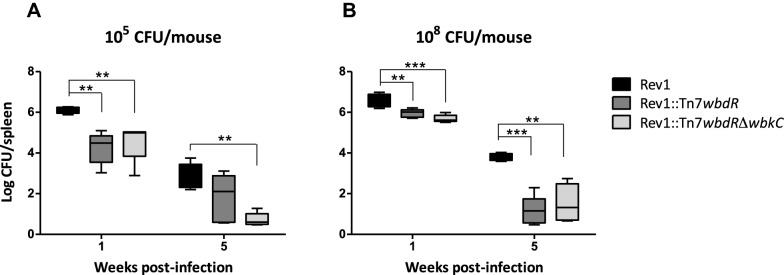
***wbdR* tagging decreases the residual virulence of Rev1.** BALB/C mice were inoculated with the indicated doses and CFU/spleen determined at post-infection weeks 1 and 5 (*p < 0.05, **p < 0.01, ***p < 0.001).

We vaccinated BALB/c mice with 10^5^ CFU/mouse (standard dose for *Brucella* S vaccines) of Rev1::Tn7*wbdR*, Rev1::Tn7*wbdR*Δ*wbkC* and Rev1 and determined CFU numbers in spleen. One week post-infection, CFU/spleen values were significantly lower in mice infected with Rev1::Tn7*wbdR* or Rev1::Tn7*wbdR*Δ*wbkC* than in those infected with Rev1 (Figure 3A). At week 5, though both Rev1::Tn7*wbdR* and Rev1::Tn7*wbdR*Δ*wbkC* also showed lower CFU, only the values of Rev1::Tn7*wbdR*Δ*wbkC* were statistically different (Figure 3A). However, attenuation with respect to Rev1 was clearly observed for both tagged vaccines at post-infection weeks 1 and 5 when the experiment was carried out with 10^8^ CFU/mouse, the dose recommended for R vaccines (Figure 3B).

### Mice vaccinated with Rev1 *wbdR* constructs can be discriminated by ELISA

**Figure 4:**
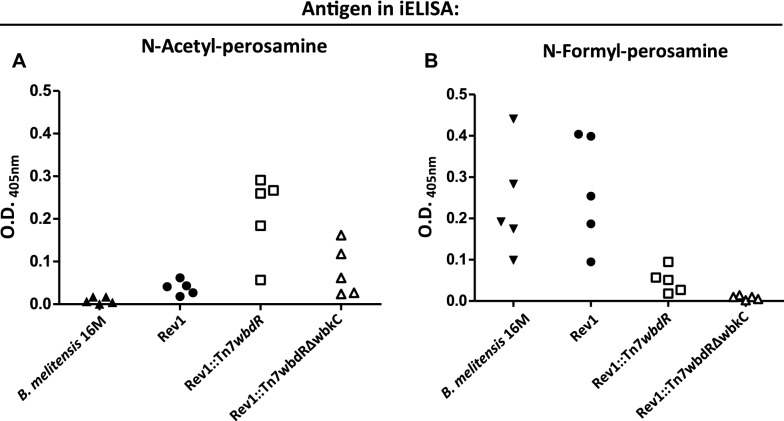
**Mice vaccinated with Rev1** ***wbdR* constructs can be discriminated by iELISA.** The sera of mice obtained 5 weeks after inoculation with Rev1, Rev1::Tn7*wbdR* or Rev1::Tn7*wbdR*Δ*wbkC* were analyzed by iELISA with N-acetyl-perosamine S-LPS (from a Tn7*wbdR*Δ*wbkC* construct of *B. abortus* 2308) (**A**) or N-formyl-perosamine S-LPS (from *B. melitensis* 16 M) (**B**). Sera from *B. melitensis* 16 M-infected mice were included as a control. The serum dilutions used was 1:20 (**A**) and 1:40 (**B**).

We then analyzed by iELISA the sera of mice vaccinated with 10^8^ CFU using first the S-LPS of Ba::Tn7*wbdR*Δ*wbkC* as antigen. As expected, and no matter the vaccine, no serum from these mice showed ELISA reactivity one week post-infection (not shown). At week 5, whereas most sera of mice inoculated with the *wbdR* constructs showed some reactivity, sera of mice infected with Rev1 or *B. melitensis* did not react (Figure 4A). Interestingly, the reverse picture was obtained in the iELISA with wild-type S-LPS as antigen: whereas mice infected with Rev1 or *B. melitensis* developed reacting antibodies, sera of mice inoculated with *wbdR* constructs displayed almost no reaction (Figure 4B) with small but consistently repeated (not shown) differences between Rev1::Tn7*wbdR* and Rev1::Tn7*wbdR*Δ*wbkC.*

### Rev1::Tn7*wbdR*Δ*wbkC* protects against *B. ovis* in the mouse model

Based on the ELISA results and on its lack of reactivity with the anti-Formyl serum (Additional file 4), we chose Rev1::Tn7*wbdR*Δ*wbkC* to study the efficacy of *wbdR* tagged vaccines against *B. ovi*s. As shown in Table 1, this vaccine conferred protection at the two doses tested. However, only the 10^8^ CFU/mouse dose (used for rough vaccines in the mouse model) conferred a protection similar to Rev1 at the standard dose (Table 1). Interestingly, even at this higher dose, the vaccine CFU remaining in the spleens was markedly lower than those of Rev1 (*p* < 0.01).

**Table 1:** **Protection induced by Rev1::Tn7*wbdRΔwbkC* against** ***B. ovis***

Vaccine (dose)	Mean ± SD of log_10_ CFU in spleen of:		Units of protection
	*B. ovis*	Vaccine	
Rev1 (10^5^)	2.45 ± 1.35^a^	2.82 ± 0.99	3.81
Rev1::Tn7*wbdR*Δ*wbkC* (10^5^)	4.47 ± 1.40	0.69 ± 0.09^c^	1.79
Rev1::Tn7*wbdR*Δ*wbkC* (10^8^)	2.16 ± 0.95^a, b^	0.90 ± 0.53^c^	4.10
PBS (unvaccinated)	6.26 ± 0.16		0.00

Statistical comparison (*n* = 5) of mean log_10_ *B. ovis* CFU/spleen: ^a^*p* < 0.001 vs. PBS (unvaccinated), ^b^*p* > 0.05 vs. Rev1 and log_10_ vaccine CFU/spleen ^c^ *p* < 0.01 vs. Rev1.

## Discussion

The results presented in this work demonstrate that the O-PS of vaccine Rev1 can be antigenically tagged by genomic insertion of *wbdR*, the acetyltransferase gene involved in the synthesis of the O-PS repeating unit of *E. coli* O157:H7. Using the mouse model, we studied the protective capacity of *wbdR* tagged Rev1 against *B. ovis* and tested an ancillary ELISA DIVA test. In previous and preliminary experiments, we examined whether *wbdR* tagged brucellae (*wbdR*Δ*wbkC*) could be used as antigen in a simple test similar to the Rose Bengal test for brucellosis. However, we observed that the modified brucellae had a marked tendency to autoagglutinate, a feature that is reminiscent of the autoagglutination typical of R brucellae [16] and shows profound cell surface modifications. For this reason, we ruled out the agglutination test and applied an iELISA using the LPS purified from a *wbdR*Δ*wbkC* construct [23] adjusted to minimize any cross-reactivity between acety-tagged and native O-PS. In this assay, we observed differences between the antibodies triggered by *wbdR* tagged vaccines when compared with those triggered by Rev1; whereas sera of mice vaccinated with Rev1::Tn7*wbdR* or Rev1::Tn7*wbdR*Δ*wbkC* showed moderate reaction, sera corresponding to the Rev1 vaccinated mice did not react. Moreover, when the iELISA with wild-type S-LPS was used, contrary to the diagnostically interfering reaction showed by Rev1, the sera of mice inoculated with *wbdR* constructs displayed almost no reaction. In a previous work, we observed that the LPS of *Brucella* cross-reacting *E. hermanii* serotypes reacts strongly with sera from infected cattle [21, 33] which are known to contain an overwhelming majority of antibodies of overlapping C specificities [34]. The O-PS of these *E. hermanii* strains contains N-acetylated perosamine in an α (1–3) and α (1–2) linkage frequency and arrangement different from those in Rev1 (i.e. *B. melitensis* biovar 1) and cross-react with *Brucella* M monoclonal antibodies in gel immunoprecipitation [33]. The O-PS in the *wbdR*Δ*wbkC* constructs does not react in ELISA with C/Y-A = M or al C/Y-A > M in ELISA [23]. These data suggest epitopic differences that, in addition to differences in the intensity of the stimulus in an experimental infection in mice and a natural infection in cattle, in the subsequent different avidity of the antibodies and in the standardization of the ELISA, could account for these apparently contradictory results. Our results suggest that the use of *wbdR* tagged Rev1 and its ancillary ELISA might be a suitable strategy to solve the DIVA problem associated with the unmodified vaccine, but it is clear that experiments in sheep are necessary for a definite assessment.

In keeping with previous studies in virulent brucellae [23], the most profound modification was obtained in Rev1::Tn7*wbdR*Δ*wbkC* and, accordingly, we tested its protective capacity in the mouse model. We observed that a 10^8^ CFU/mouse dose was necessary to reach the protection levels obtained with Rev1 at 10^5^ CFU/mouse. The recommended dose for brucellosis R vaccines is also 10^8^ CFU/mouse [35] and, like the autoagglutination commented above this suggests surface similarities between the Rev1::Tn7*wbdR*Δ*wbkC* construct and R brucellae. The lack of O-PS in the latter increases the exposure of lipid A-core and Omp charged groups and it has been postulated that the OP-S acts as a negative modulator of unspecific interactions of these bacteria with host cells, thus favoring invasion for specific routes [18]. On these bases, it can be hypothesized that either the N-acetyl-perosamine O-PS cannot replace the N-formyl-perosamine with regards to surface physicochemical properties and/or that the shorter O-PS of Rev1::Tn7*wbdR*Δ*wbkC* constructs does not provide enough steric hindrance to the Omps and the lipid A-core section, as suggested by its sensitivity to the R/C phage. These surface changes may account for our observation that, when tested for the residual virulence (Figure 3), we found that the CFU counts/spleen of Rev1::Tn7*wbdR*Δ*wbkC* were lower than those of Rev1 even at a dose one thousand times higher, a result in agreement with the low numbers of CFU remaining in the spleens in the vaccination experiments (Table 1). Taken together, these results in mice suggest that Rev1::Tn7*wbdR*Δ*wbkC* could be not only a DIVA vaccine but also a vaccine with reduced residual virulence in the host. Research is in progress to evaluate serological response and protective capacity against *B. ovis* of Rev1::Tn7*wbdR*Δ*wbkC* in sheep.

## Supplementary information

**Additional file 1. Bacterial strains and plasmids.**

**Additional file 2. Primers.**

**Additional file 3. Differential characteristics of species of the genus *Brucella* and mutants.**

**Additional file 4. Insertion of *wbdR* into Rev1 genome modifies the epitopic structure of the vaccine.**
