# Correction to: Rev1 *wbdR* tagged vaccines against *Brucella ovis*

**DOI:** 10.1186/s13567-020-00752-6

**Published:** 2020-02-19

**Authors:** Beatriz Aragón‑Aranda, María Jesús de Miguel, Estrella Martinez‑Gomez, Amaia Zuñiga‑Ripa, Miriam Salvador‑Bescós, Ignacio Moriyón, Maite Iriarte, Pilar M. Muñoz, Raquel Conde‑Alvarez

**Affiliations:** 1 Instituto de Salud Tropical (ISTUN), Instituto de Investigación Sanitaria de Navarra (IdiSNA) and Dpto. de Microbiología y Parasitología, Universidad de Navarra, c/Irunlarrea 1, 31008 Pamplona, Spain; 2 Unidad de Producción y Sanidad Animal, Instituto Agroalimentario de Aragón-IA2 (CITA-Universidad de Zaragoza), Av. Montañana 930, 50059 Saragossa, Spain

## Correction to: Vet Res (2019) 50:95 10.1186/s13567-019-0714-3

In the original publication of this article [1], the corresponding author points out Pilar M. Muñoz and Raquel Conde‑Alvarez contributed equally to this work.
