# Supplementary material for: Rev1 wbdR tagged vaccines against Brucella ovis
Source: Vet Res. 2019 Nov 15;50:95. doi: 10.1186/s13567-019-0714-3 (PMC6858679; doi:10.1186/s13567-019-0714-3)
Supplement: Supplementary file 1 — Additional file 1. Bacterial strains and plasmids. [file 13567_2019_714_MOESM1_ESM.doc]

Additional File 1. Bacterial strains and plasmids.

| **Strains** | **Characteristics** | **Reference** |
| --- | --- | --- |
| ***Brucella melitensis*** |  |  |
| Rev1 | *B. melitensis* Rev1 vaccine reference strain. | CITA collection |
| Rev1::Tn7*wbdR*km^R^ | Rev1 vaccine with miniTn7 transposon, carrying the *wbdR* gene of *E. coli* O157:H7 inserted in the chromosome. | This work |
| Rev1::Tn7*wbdR* | Rev1 vaccine with miniTn7 transposon, carrying the *wbdR* gene of *E. coli* O157:H7 inserted in the chromosome. It carries an internal deletion in the kanamycin gene (Δ*Km*_Δ2-261_). | This work |
| Rev1::Tn7*wbdR*Δ*wbkC* | Rev1 vaccine with miniTn7 transposon, carrying the *wbdR* gene of *E. coli* O157:H7 inserted in the chromosome. It carries an internal deletion in the kanamycin gene (Δ*Km*) and *wbkC* gene (Δ*wbkC*_Δ29-211_). | This work |
| ***Brucella ovis*** |  |  |
| *B. ovis* PA | Challenge strain used in *B. ovis* vaccine studies; virulent. | [1] |
| BoPA-Km^R^ | *B. ovis* PA with mini Tn7 transposon (pUC18R6KT-miniTn7T-Km). Challenge strain. | This work |
| ***Escherichia coli*** |  |  |
| S17-1λpir | Mating strain with plasmid RP4 inserted into the chromosome. | [2] |
| SM10 λpir | *th*-1 *thr leu tonA lacY supE, recA*::RP4-2-Tc::Mu KmR (λpir). | [3] |
| HB101 | F - *hsdS20 recA13ara -14 proA2 lacY1galK2 rpsL20xyl-5mtl-*1 *supE44.* | [4] |
| β2150 | F´lacZΔM15 *laclq* *pro*A+B+ *thr*B1004 *pro thi strA hsds ΔdapA::erm (Ermr) pir.*  *E. coli* deficient in the Diaminopimelic Acid (DAP) synthesis | [5] |
| **Plasmids** |  |  |
| pRK2013 | Helper vector containing tra and mob genes | [6] |
| pTNS2 | Plasmid expressing *tns*ABCD from P*lac.* ApR | [7] |
| pUC18R6KT-miniTn7T-Km | pUC18R6KT-miniTn7T-Km with Km cassette | [8] |
| pYRI-27 | 727-bp of *E. coli* O157:H7 chromosomal DNA containing the *wbdR* ORF and 300-bp upstream, cloned into pUC18R6KT-miniTn7T-Km | [9] |
| pRCI-65 | pNPTS138Cm^R^ carrying a *Km* deletion allele | [9] |
| pYRI-31 | pJQKm suicide plasmid carrying a *wbkC* deleted allele | [9] |

**BIBLIOGRAPHY**

1. Blasco JM, Marín C, Jiménez de Bagüés MP, Barberán M (1993). Efficacy of *Brucella suis* strain 2 vaccine against *Brucella ovis* in rams. Vaccine. 11:1291–4. doi:10.1016/0264-410X(93)90097-H.

2. Simon R, Priefer U, Pühler A (1983). A Broad Host Range Mobilization System for *In Vivo* Genetic Engineering: Transposon Mutagenesis in Gram Negative Bacteria. Bio/Technology. 1:784–91. doi:10.1038/nbt1183-784.

3. Miller VL, Mekalanos JJ (1988). A novel suicide vector and its use in construction of insertion mutations: osmoregulation of outer membrane proteins and virulence determinants in *Vibrio cholerae* requires toxR. J Bacteriol. 170(6): 2575–83.

4. Sambrook J, Fritsch EF, Maniatis T (1989). Molecular cloning: a laboratory manual. Mol cloning a Lab manual.; Ed. 2.

5. Dehio C, Meyer M (1997). Maintenance of broad-host-range incompatibility group P and group Q plasmids and transposition of Tn5 in Bartonella henselae following conjugal plasmid transfer from Escherichia coli. J Bacteriol. 179:538–40. doi:10.1128/jb.179.2.538-540.1997.

6. Figurski DH, Helinski DR (1979). Replication of an origin-containing derivative of plasmid RK2 dependent on a plasmid function provided in trans. Proc. Nati. Acad. Sc 76(4): 1648–52.

7. Choi K-H, Gaynor JB, White KG, Lopez C, Bosio CM, Karkhoff-Schweizer RR, et al. A Tn7-based broad-range bacterial cloning and expression system. Nat Methods. 2005;2:443–8. doi:10.1038/nmeth765.

8. Llobet E, March C, Gimenez P, Bengoechea JA (2009). *Klebsiella pneumoniae* OmpA Confers Resistance to Antimicrobial Peptides. Antimicrob Agents Chemother. 53:298–302. doi:10.1128/AAC.00657-08.

9. Martínez-Gómez E, Ståhle J, Gil-Ramírez Y, Zúñiga-Ripa A, Zaccheus M, Moriyón I, Iriarte M, Wildmalm G, Conde-Álvarez R (2018). Genomic Insertion of a Heterologous Acetyltransferase Generates a New Lipopolysaccharide Antigenic Structure in *Brucella abortus* and *Brucella melitensis*. Front Microbiol. 9:1092. doi:10.3389/fmicb.2018.01092.
