# Supplementary material for: Rev1 wbdR tagged vaccines against Brucella ovis
Source: Vet Res. 2019 Nov 15;50:95. doi: 10.1186/s13567-019-0714-3 (PMC6858679; doi:10.1186/s13567-019-0714-3)
Supplement: Supplementary file 2 — Additional file 2. Primers. [file 13567_2019_714_MOESM2_ESM.docx]

**Additional file 2 Primers**

| **Primers** | **Sequence 5´- 3´** | **Used to verify** |
| --- | --- | --- |
| *GlmS*_B | GTCCTTATGGGAACGGACGT | *wbdR* insertion |
| Ptn7-R | CACAGCATAACTGGACTGATT | *wbdR* insertion |
| Ptn7-L | ATTAGCTTACGACGCTACACCC | *wbdR* insertion |
| *RecG* | TATATTCTGGCGAGCGATCC | *wbdR* insertion |
| *Km*R-F1 | AGGAAGCGGAACACGTAGAA | *km* deletion |
| *Km*R-R4 | TGGTCCATATGAATATCCTCCTTA | *km* deletion |
| *wbkC*-F1 | AGGTGGCGACAAACGAATAA | *wbkC* deletion |
| *wbkC*-R4 | tctgaactcggctggatgac | *wbkC* deletion |
