# Supplementary material for: Rev1 wbdR tagged vaccines against Brucella ovis
Source: Vet Res. 2019 Nov 15;50:95. doi: 10.1186/s13567-019-0714-3 (PMC6858679; doi:10.1186/s13567-019-0714-3)
Supplement: Supplementary file 3 — Additional file 3. Differential characteristics of species of the genus Brucella and mutants. [file 13567_2019_714_MOESM3_ESM.docx]

**Additional file 3 Differential characteristics of species of the genus *Brucella* and mutants.**

|  |  | **Growth on dye media** | | | | | | | | | | | |  |  |  |
| --- | --- | --- | --- | --- | --- | --- | --- | --- | --- | --- | --- | --- | --- | --- | --- | --- |
|  |  | Thionine (µg/mL) | | | | | | Fuchsin (µg/mL) | | | | Safranin (µg/mL) | |  |  |  |
|  |  | 10 | | 20 | | 40 | | 10 | | 20 | | 100 | | **Lysis by phages^a^**  **(RTD)^b^** | | |
| **Strain** | **Urease activity** | CO_2_ | without CO_2_ | CO_2_ | without CO_2_ | CO_2_ | without CO_2_ | CO_2_ | without CO_2_ | CO_2_ | without CO_2_ | CO_2_ | without CO_2_ | Tb | Wb | R/C^c^ |
| *B.melitensis* 16M | + | + | + | + | + | - | - | + | + | + | + | + | + | - | - | - |
| Rev1 | + | + | + | + | - | - | - | + | + | + | + | + | - | - | - | - |
| Rev1::Tn7*wbdR* | + | + | + | + | + | + | - | + | + | + | + | + | - | - | - | -2 |
| Rev1::Tn7*wbdR*∆*wbkC* | + | + | + | + | + | - | - | + | + | + | + | + | - | - | - | -2 |
| *B.ovis* PA | - | + | - | + | - | + | - | - | - | - | - | - | - | - | - | -5 |

1. Phages: Tbilisi (Tb), Weybridge (Wb) and R/C
2. RTD: routine test dilution
3. The numbers correspond to the dilutions of the phages to which the strains are lysed
