# Supplementary material for: Rev1 wbdR tagged vaccines against Brucella ovis
Source: Vet Res. 2019 Nov 15;50:95. doi: 10.1186/s13567-019-0714-3 (PMC6858679; doi:10.1186/s13567-019-0714-3)
Supplement: Supplementary file 4 — Additional file 4. Insertion of wbdR into Rev1 genome modifies the epitopic structure of the vaccine. [file 13567_2019_714_MOESM4_ESM.docx]

**Additional file 4** **Insertion of *wbdR* into Rev1 genome modifies the epitopic structure of the vaccine**^1^

| Strain | Reactivity with serum specific for: | | |  |  |
| --- | --- | --- | --- | --- | --- |
|  | Formyl-Acetyl | Acetyl | Formyl | |  |
| *B. melitensis* 16M | + | - | + | | |
| Rev1 | + | - | + | | |
| Rev1::Tn7*wbdR* | + | + | + | | |
| Rev1::Tn7*wbdR*Δ*wbkC* | + | + | - | | |

^1^ Aliquots of each bacterial suspension (4-6 colonies resuspended in 25 µL of saline) were mixed with an equal volume of *Staphylococci* sensitized with the indicated sera and the reaction scored as positive (+) or negative (-) after two minutes of incubation at room temperature.
